# Approaches for Curriculum and Assessment in Leader and Leadership Education and Development Programs in American Medical Schools

**DOI:** 10.15694/mep.2018.0000244.1

**Published:** 2018-10-25

**Authors:** Erin Barry, Neil Grunberg, Hannah Kleber

**Affiliations:** 1 Uniformed Services University of the Health Sciences

**Keywords:** Leader and Leadership Education and Development, Leaders, Leadership, Medical Leader and Leadership Education and Development, LEAD

## Abstract

This article was migrated. The article was marked as recommended.

**Problem:** There is a growing call to add leader and leadership education to undergraduate medical education (UME). Yet, there currently are no established standards, competencies, curricula, or requirements for UME leader and leadership education and development (LEAD) programs. The Uniformed Services University of the Health Sciences (USU) F. Edward Hébert School of Medicine LEAD program hosts annual Summit and Working Group meetings to address issues and to share experiences about LEAD programs.

**Approach:** Based on survey results following the 2017 USU LEAD Summit, working group participants reported that the meeting was valuable, should be repeated, and should address the specific topics of curriculum and assessment. Therefore, the 2018 Summit’s goal was for participants to share experiences, ideas, and ways forward regarding leader and leadership curricula and assessment measures for UME. Themes from working groups were compiled and reported.

**Outcomes:** Themes within LEAD curriculum include: (1) what to teach: relevant knowledge, skills, and abilities/attitudes (KSA) for specific topics; (2) when to teach: a life-cycle program woven through UME into graduate medical education and beyond; and (3) how to teach: near peers, development of mentors, and near-term, practical applications of skills. Themes within LEAD assessment include: (1) what to assess: alignment with program goals and curriculum within a positive culture of assessment and trust; (2) when to assess: occur at times that are consistent with the learning objectives and curriculum to provide information on incremental “growth” of students and the program; (3) how to assess: use formative and summative, qualitative and quantitative measures that are reliable and valid.

**Next steps:** Based on feedback from working group participants at the 2018 Summit, the USU LEAD team will host a third Summit in April 2019 focusing on leader and leadership education and development across the healthcare work force life cycle.

## Problem

Undergraduate medical education (UME) requirements already are extensive and the continuous introduction of new procedures, instruments, medications, and record systems keep adding more to learn. In addition, there is a growing clarion call to add leadership education to UME ( Webb *et al.*, 2014; Frich *et al.*, 2015; Neeley, Clyne and Resnick-Ault, 2017; Grunberg *et al.*, 2018). According to the AAMC, leadership is “the most critical component for success (Association of American Medical Colleges).” Yet, there currently are no established standards, competencies, curricula, or requirements for UME leader and leadership education and development (LEAD) programs (where leader refers to human capital and leadership refers to social capital ( Day, 2001)). An increasing number of medical schools are offering LEAD programs ( Hargett *et al.*, 2017; Barry *et al.*, 2018; Hopkins *et al.*, 2018) and many are sharing information relevant to their LEAD curriculum and assessment instruments. Among these efforts, the Uniformed Services University of the Health Sciences (USU) F. Edward Hébert School of Medicine LEAD program - that trains healthcare professionals, officers, and leaders for the United States Air Force, Army, Navy, and Public Health Service - hosts annual Summit and Working Group meetings (beginning in 2017) to address issues and to share experiences about LEAD programs.

## Approach

The annual USU LEAD Summit and Working Group meetings are convened for faculty and staff from U.S. Schools of Medicine, service academies, and related professional organizations to address issues relevant to health workforce leader and leadership education and development. At the 2017 Summit, participants shared opinions, experiences, and best practices regarding medical school leadership programs ( Grunberg *et al.*, 2018). Based on survey results following the 2017 Summit, participants reported that the meeting was valuable, should be repeated, and should address the specific topics of curriculum and assessment. Therefore, the 2018 Summit’s goal was for working group participants to share experiences, ideas, and ways forward regarding leader and leadership curricula and assessment measures for undergraduate medical education (UME).

The Summit began with an overview of the schedule, purpose, and introductions of participants. The morning session focused on small working group discussions of curriculum followed by a networking opportunity and then a plenary discussion of major points raised in the small group discussions. The afternoon session focused on small working group discussions of assessment followed by further networking and then a plenary discussion of major points raised in the working groups. The meeting concluded with a large group discussion of next steps.

Similar to the 2017 Summit, ( Grunberg *et al.*, 2018) working group participants included individuals from public and private U.S. medical schools engaged in leader and leadership education and development. Working group participants also included individuals from: United States Military Academy (USMA), United States Naval Academy (USNA), United States Naval War College, and Marine Corps University. In addition, representatives from the Association of American Medical Colleges (AAMC) and Veterans Administration (VA) participated (see Table 1).

**Table 1.** List of working group participants and their affiliations.

Working Group Participants	Institution
Gene Andersen, MA	United States Naval War College
Erin Barry, MS *	Uniformed Services University
Eric Bean, DO, MBA	University of South Florida, Lehigh Valley
Andrew Bergemann, PhD	University of Texas at Austin
Mary Brueggemeyer, MD	Uniformed Services University
Ronald Cervero, PhD	Uniformed Services University
Matthew Clark, PhD, PMP	United States Army
Joe Doty, PhD	Duke University
Kathryn Eklund, MS	Uniformed Services University
David Fessell, MD *	University of Michigan
Tanja Fessell, MS	University of Michigan
Alexander Galifianakis, MD	Uniformed Services University
Craig Goolsby, MD, MEd	Uniformed Services University
Neil Gunberg, PhD *	Uniformed Services University
Nathan Hudepohl, MD, MS, MPH *	Brown University
Nancy Hueppchen, MD, MSc	Johns Hopkins University
Hannah Kleber, BA *	Uniformed Services University
Shari Lawson, MD	Johns Hopkins University
Lauren Mackenzie, PhD	Marine Corps University
Ronald Massey, MA, MPA	Veterans Administration
Christopher Mattos, MA	United States Military Academy
John McManigle, MD	Uniformed Services University
Katherine McOwen, MSEd	Association of American Medical Colleges
David Musick, MD	Virginia Tech University
Francis O’Connor, MD, MPH	Uniformed Services University
Kathleen Ogle, MD	George Washington University
Louis Pangaro, MD	Uniformed Services University
Lisa Perla, MA	Uniformed Services University; Graduate School of Nursing
Penny Pierce, PhD	Uniformed Services University; Graduate School of Nursing
Joann Quinn, PhD	University of South Florida
Stacey Rizza, MD	Mayo Clinic
Douglas Robb, DO, MPH	Uniformed Services University
Carol Romano, PhD, RN	Uniformed Services University; Graduate School of Nursing
Eric Schoomaker, MD, PhD	Uniformed Services University
Amy Smith, PhD	University of South Florida, Lehigh Valley
Steven Spector, PhD	University of South Florida
Erin Sullivan, PhD	Harvard University
Dean Taylor, MD	Duke University
Suzanne Templer, DO *	Nova Southeastern University
Joslyn Vaught, BS	Mayo Clinic
David Wallace, PhD	United States Naval Academy
Joseph Weistroffer, MD	Western Michigan University
Dean Winslow, MD	Stanford University
Melanie Wong-Dodge, MBA	United States Military Academy Preparatory School

* indicates working group facilitators

There were six working groups composed of six to seven participants representing different organizations, demographics, and perspectives (e.g., physicians, nurses, psychologists, health profession educators, researchers, administrators). Each group had a facilitator who was given a list of questions to help guide the discussions (see Tables 2 and 3). Both morning and afternoon small group sessions lasted 60 minutes; networking sessions were 30 minutes; large group discussions were 30 minutes.

**Table 2.** List of Curriculum questions for facilitators to guide working group discussion

• What are your top examples of curricular content? • Where do you believe the leadership curriculum best fits within the overall medical school curriculum? • How do you sequence leadership curriculum into medical school curriculum (pre-clinical and clinical)? • Do you envision your leadership program for all students or for some students? • Do you primarily use leadership activities (that may or may not be related to other leadership activities)? • Do you find that your leadership curriculum is supported by the rest of the student experience? • What educational methodologies do you use most in your program (e.g., small group, didactic)? • What resources are required (or do you envision having) for your session? • What leadership sessions have been most well-received by your students? • What experiences have you had with sessions that were not received well? • Why did you believe it would be well-received? What were the unanticipated problems with this session? • What feedback have you received from students regarding curriculum topics and method delivery? • What are student views on leadership in the medical school curriculum? • Have you attempted any faculty development? What approaches have you used (if any) for fostering faculty development in leadership?

**Table 3.** List of Assessment questions for facilitators to guide working group discussion

• What do you currently assess? • What are you trying to assess? • Is assessment a requirement for your current program? If so, in what way? • How do you currently assess? • Students • Faculty • Overall Program/curriculum • Do you currently have any specific tools that you use to assess (i.e., quiz questions, observations, surveys, qualitative or quantitative data)? • Are your assessment goals short-term or long-term? • What role does assessment play in your current program? • Have you been able to measure the effect of your leadership program (either before or after your students have left)?

Facilitators recorded the main points from each discussion and submitted these notes to the Summit organizers. Three coders (who were small group facilitators - a senior faculty member, a junior faculty member, and an education specialist) independently analyzed the submitted notes from all six working groups. Coders were instructed to identify three to five themes into which the individual points regarding curriculum could be categorized and three to five themes into which the individual points regarding assessment could be categorized. The three coders met to compare and discuss their initial classification results. There was complete agreement among the three coders that the points raised in all working groups could be classified into what, when, and how for curriculum and for assessment. There was substantial agreement among the three coders with regard to the classification of the many individual points recorded by facilitators; differences were resolved by discussion and majority consensus among the three coders. The outcomes (see below) reflect these findings.

## Outcomes

### Curriculum

***What to teach**.* All working group discussions indicated that LEAD curriculum should teach knowledge, skills, and abilities/attitudes (KSA) relevant to effective and successful performance as a leader. KSA topics most frequently mentioned were: professional identity as healthcare leaders, medical ethics, emotional intelligence, and teamwork.

***When to teach**.* LEAD must be approached as a life-cycle program and should start early - even as early as high school and undergraduate college education. This life-cycle approach should be woven through UME and continued during graduate medical education (GME) and beyond in a coherent manner. The life-cycle approach should guide when particular KSAs are taught.

***How to teach**.* The working groups indicated the importance of: the inclusion of near peers (as small group facilitators, discussion leaders, etc.) in teaching; (2) development of mentors (including faculty and peers as mentors for students); and (3) providing relevant, near-term, practical applications of KSAs.

### Assessment

***What to assess**.* It is important that program goals, curricula, and assessment are well aligned. A positive and supportive culture of assessment and trust needs to be fostered, cultivated, and emphasized. Establishing positive culture and trust with assessment takes thoughtful effort and time to implement.

***When to assess**.* Assessment measures should occur at times that are consistent with the learning objectives and curriculum and should assess students, instructors, and programs by students, instructors, and stakeholders. There should be consideration of where each student is in the LEAD education life-cycle for meaningful interpretation of current status and incremental “growth” of individual students and of the program. It is important to have buy-in from students, instructors, and stakeholders throughout the education life-cycle (UME, GME, and beyond).

***How to assess**.* Assessments should use formative and summative, qualitative and quantitative measures that are reliable and valid. These measures should be based on where the student is within the life-cycle of education and development. Additionally, Kirkpatrick levels of training evaluation (Reaction; Learning; Behavior; Results) are useful to assess students and programs ( Kirkpatrick and Kirkpatrick, 2016). It is critical to include faculty development so that instructors understand the program’s philosophy, curriculum, goals, and assessment strategies, and how to optimally assess students.

## Next Steps

### Curriculum

***What to teach**.* LEAD programs should identify specific KSAs relevant to professional identity as healthcare leaders, medical ethics, emotional intelligence, and teamwork, and design sessions to address them.

***When to teach**.* A life-cycle approach would allow programs to teach KSAs in a laddered manner to allow students to gain better understanding and application of the KSAs. This approach would allow students to incorporate skills into practical applications.

***How to teach**.* Students learn in a variety of ways with the greatest impact derived from hands-on experiences and relevant, practical examples in educational sessions. Near peer facilitators and sound mentor relationships can enhance students’ leadership development.

### Assessment

***What to assess**.* Establishing a culture of assessment that emphasizes mutual respect, trust, and meaningful critiques (i.e., indicating successes and what to continue as well as what to improve) are essential to assess students, faculty, and programs.

***When to assess**.* Regular and expected feedback provide measures of incremental growth and remind students of the value of the KSAs. Additionally, it is important to evaluate the incremental growth of the program to improve the experience for students.

***How to assess**.* Reliable, valid assessment measures that are aligned with the goals of the program and which span UME to provide information for students about their incremental growth are essential. Kirkpatrick levels of training evaluation will allow programs to assess students, faculty, as well as the effectiveness of the program.

### Future Summit

Based on feedback from working group participants at the 2018 Summit, the USU LEAD team will host a third Summit in April 2019. The 2019 LEAD Summit will focus on leader and leadership education and development across the healthcare work force life cycle.

## Take Home Messages

• Working group meetings focused on medical leader and leadership education and development (LEAD) are valuable.
• LEAD curricula should consider what, when, how; more specifically: relevant KSAs; life-cycle programs woven throughout UME, GME, and beyond; near peers, mentor development, and near-term, practical applications.
• LEAD assessments should consider what, when, how; more specifically: alignment with program goals and establishment of a positive assessment culture; occur consistently with the learning objectives and curriculum to provide information on incremental “growth” of students and the program; use of formative and summative, qualitative and quantitative measures that are reliable and valid.

## Notes On Contributors

**Erin S. Barry**, MS, is a Research Assistant Professor in the Department of Military & Emergency Medicine (MEM), Research Associate for Leader and Leadership Education and Development (LEAD), and doctoral student in the Health Professions Education Program, Uniformed Services University of the Health Sciences (USU). ORCID: https://orcid.org/0000-0003-0788-7153.

**Neil E. Grunberg**, PhD, is a Professor in MEM and Director of Research & Development LEAD, USU.

**Hannah G. Kleber**, BA, is an Education Specialist for LEAD, USU.
