# Supplementary material for: Approaches for Curriculum and Assessment in Leader and Leadership Education and Development Programs in American Medical Schools
Source: MedEdPublish (2016). 2018 Oct 25;7:244. [Version 1] doi: 10.15694/mep.2018.0000244.1 (PMC10711993; doi:10.15694/mep.2018.0000244.1)
Supplement: Peer review history

# Reviewer report: Reviewer response for version 1

**DOI:** 10.21956/mep.19714.r29504

**Published:** 2018-11-08

**Authors:** P Ravi Shankar

**Affiliations:** 1 American International Medical University

This review has been migrated. The reviewer awarded 4 stars out of 5 I enjoyed reading this report about the 2018 LEAD summit. Leadership education is important for doctors but the primary focus and role of a leader may differ according to the vision and mission of the university/institution. The working groups’ deliberations provide inputs regarding leadership education and assessment. Looking at Table 1 I note that participation was restricted to certain US medical schools. This could be a limitation. The summit was held at the Uniformed Services University and the majority of participants were from the military. This could have an influence on their perspectives about leadership and influenced the deliberations. The working groups provide a general perspective on teaching and assessing leadership skills in medical education. This brief paper will be of interest to medical educators in a variety of settings. The context and the vision and mission of the school and the region or country could and should influence the leadership education curriculum.

Reviewer Expertise:

NA

No decision status is available

# Reviewer report: Reviewer response for version 1

**DOI:** 10.21956/mep.19714.r29503

**Published:** 2018-10-26

**Authors:** Tan Nguyen

**Affiliations:** 1 Deakin University

This review has been migrated. The reviewer awarded 4 stars out of 5 This work offers an excellent example of cross-institutional collaborations on key important issues relevant to medical education, which could be adopted by other health disciplines. I think there will be still a substantial body of work to be done to identify/manage the expectations and develop a program that will address the learning needs of the delegates at the Summit. An evaluation of the LEAD program could be developed to articulate how the program impacts on learning. I would be great to discuss how these themes identified may or may not be similar to those reported in the medical education literature on leadership.

Reviewer Expertise:

NA

No decision status is available

# Reviewer report: Reviewer response for version 1

**DOI:** 10.21956/mep.19714.r29501

**Published:** 2018-10-26

**Authors:** Richard Hays

**Affiliations:** 1 James Cook University

This review has been migrated. The reviewer awarded 4 stars out of 5 This paper addresses a topical issue that should be of interest to many readers. We are in an era when leadership is spoken of often as a need to lead health care through challenging times. A common response is to teach and assess leadership, often from the beginning of medical education. While this is sound, in principle, it opens big questions, such as 'how much more can we fit into the curriculum', 'how does this integrate with learning clinical medicine', and 'how do we assess something that is so hard to measure?'. Some useful resources are available, such as the Leadership Development Framework in the UK, and this document will be another useful resource. However, many approaches and frameworks assume that medical doctors will be the leaders and in fact can be from a young age with little or no preparation. At risk of sounding like an 'over the hill' red wine, my experience has shown that leadership develops with experience, based on reflection, self-awareness, humility, values, respectful communication and learning from mistakes. I am not sure how this journey can be shortened, but to me that is the main purpose of leadership frameworks. Start slow, with awareness and discussing examples of strong, sound and perhaps just different leadership styles. The best time to expand may be the late undergraduate/early GME periods, when individuals often feel least empowered, with leadership experience, reflection, feedback and assessment in-context. I worry that we may be asked to deliver another linear, milestone-assessed curriculum theme, with teaching and assessment methods that are not well suited to this task. This may be too important a topic to risk engendering in early career doctors a sense of: 'leadership? passed that, I deserve promotion'. What I particularly enjoyed about this paper is that the authors show awareness of these concerns, but leave open how to address them beyond principles. More work is needed to work out the details of just what, how and when to teach and assess the elements of leadership, which would see me award the extra star. Perhaps the next stage in the journey?

Reviewer Expertise:

NA

No decision status is available

# Reviewer report: Reviewer response for version 1

**DOI:** 10.21956/mep.19714.r29502

**Published:** 2018-10-25

**Authors:** Felix Silwimba

**Affiliations:** 1 University of Lusaka

This review has been migrated. The reviewer awarded 5 stars out of 5 This is a very good report of LEAD in UME. I only wish low and middle income could use a similar approach in addressing the challenges of UME. The take home messages are clear and well-articulated.

Reviewer Expertise:

NA

No decision status is available
